# Pneumomediastinum and Bilateral Pneumothoraces Causing Respiratory Failure after Thyroid Surgery

**DOI:** 10.1155/2017/8206970

**Published:** 2017-04-24

**Authors:** Michael Koeppen, Benjamin Scott, Joseph Morabito, Matthew Fiegel, Tobias Eckle

**Affiliations:** ^1^Anesthesiology, Ludwig Maximilian University of Munich, Munich, Germany; ^2^Anesthesiology, University of Denver School of Medicine, Aurora, CO, USA

## Abstract

We report the first case of severe respiratory failure after thyroid surgery requiring venovenous extracorporeal membrane oxygenation (vvECMO). The patient was a 41-year-old woman with metastatic thyroid cancer. She underwent thyroidectomy, including left lateral and bilateral central neck dissection. During surgery, the patient developed pneumomediastinum with bilateral pneumothoraces. Despite early treatment with bilateral chest tubes and no evidence of a tracheal perforation, the patient developed severe respiratory failure after extubation on the intensive care unit. Because pneumothorax and pneumomediastinum might be more common than reported, and considering increasing cases of thyroid surgery, staff should remain vigilant of pulmonary complications after thyroid surgery.

## 1. Introduction

The incidence of thyroid cancer has more than tripled since 1975 and, in women, it is the cancer with the fastest-growing number of new cases [1]. The treatment includes thyroidectomy, a very common surgical procedure with approximately 100,000 cases each year in the United States [2]. In fact, a recent study among patients in the United States diagnosed with thyroid cancer from 1974 to 2013 found that the overall incidence of thyroid cancer increased by 3% annually, with increases in the incidence and mortality rate [3]. These findings show a true increase in the occurrence of thyroid cancer in the United States. Inevitably, this may increase the number of complications from thyroid surgery and the number of anesthesiologists caring for patients undergoing thyroid surgery. Complications considered “rare” now might become more common in the future. Here, we report a patient who developed bilateral pneumothoraces and pneumomediastinum during thyroid surgery with neck dissection. After postsurgical extubation, the patient deteriorated. The bilateral pneumothoraces worsened despite bilateral chest tubes, ultimately leading to severe respiratory failure that required ECMO treatment. Rapid initiation of therapy facilitated a fast recovery and the patient was discharged. Following a thorough literature review, we identified only a few published reports of pneumothorax after thyroid surgery. However, one series of 300 cases suggests a higher incidence rate [4].

## 2. Case Description

**Figure 1:**
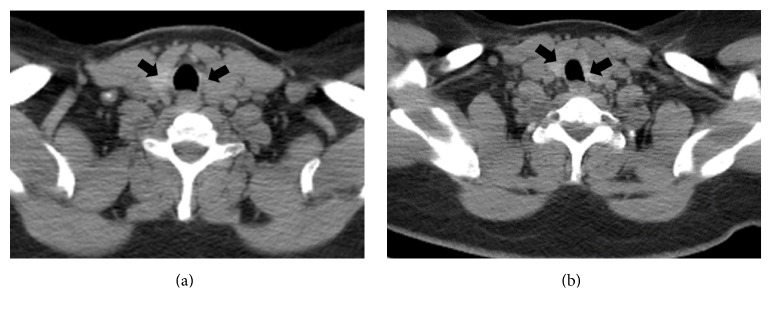
Preoperative CT scan of the neck. The preoperative CT scan of the neck shows no abnormalities. Shown are two different levels (a, b). Black arrows indicate thyroid tissue.

A 41-year-old, 75 kg, 150 cm (body mass index of 33), woman with thyroid cancer metastatic to the bilateral neck underwent thyroidectomy, including left lateral and bilateral central neck dissection. No chemotherapy or radiation therapy was employed as an adjunctive prior to surgery. Her medical history included well-controlled arterial hypertension and diabetes mellitus type 2. She was a nonsmoker and denied any history of cardiac or pulmonary disease. The physical exam prior to surgery was unremarkable. The initial CT scan of the neck showed no abnormalities of the trachea (Figure 1). The preoperative vital signs were as follows: blood pressure 121/81 mmHg, heart rate 91/min, respiratory rate of 16/min, temperature 37.4 Celsius, and a SpO_2_ of 96% on room air.

Standard anesthesia monitoring (ECG, SpO_2_, ETCO_2_, and NIBP [IBP added after 4 hours of surgery]) was established and maintained throughout the procedure. After intravenous pretreatment with midazolam 2 mg and fentanyl 0.15 mg, general anesthesia was induced using propofol 200 mg, followed by 80 mg succinylcholine to facilitate intubation. We maintained anesthesia using desflurane inhalation and remifentanil infusion (0.2 *μ*g/kg/min). To minimize the risk of recurrent laryngeal nerve damage, we used an electromyogram endotracheal tube (EMG-ETT, Medtronic), placed using a video laryngoscope and inflated the cuff to a pressure of 25 cm H_2_O. Then, guaranteed volume, pressure-controlled ventilation was established and we maintained the end-tidal CO_2_ (ETCO_2_) levels at 35 mmHg with tidal volumes (TV) of 350 ml and a respiratory rate of 15/min. The adjustable pressure limiting valve was set to 5 cm H_2_O and average peak inspiratory pressures (PIP) and mean airway pressures (AP mean) were 22 cm H_2_O and 12 cm H_2_O, respectively.

**Figure 2:**
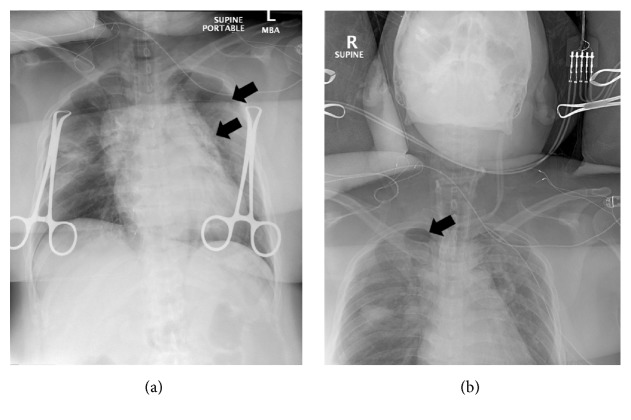
X-rays during surgery showing pneumomediastinum and pneumothorax. First intraoperative chest X-ray revealed a small left-sided pneumothorax and a mild pneumomediastinum ((a) black arrows). Second intraoperative chest X-ray found that the left pneumothorax had decreased in size; however, a new right pneumothorax had developed ((b) black arrows).

**Figure 3:**
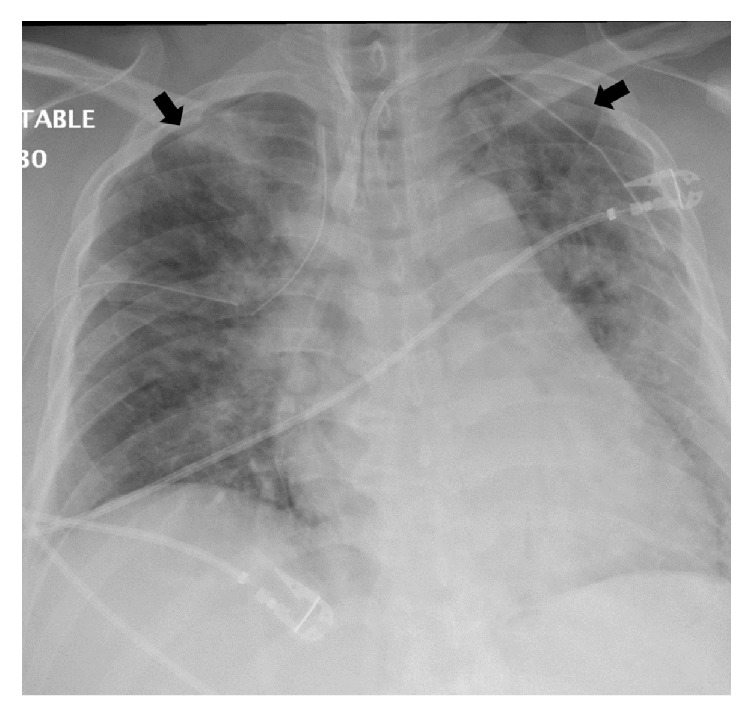
X-ray on postoperative day 1 after extubation. The patient was extubated on postoperative day 1 as he met extubation criteria. Shown is the X-ay after extubation, indicating that the bilateral pneumothoraces had increased despite bilateral chest tubes.

**Figure 4:**
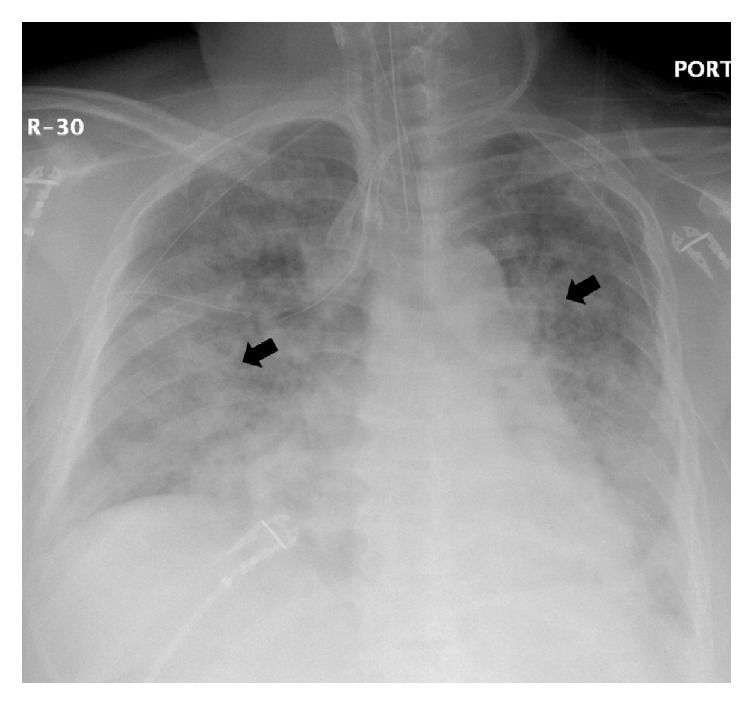
Respiratory failure on postoperative day 2. Shown is the chest X-ray with bilateral patchy opacities (black arrows) after urgent reintubation. Together with a normal left ventricular function and poor oxygenation (PaO_2_ 60 mmHg, FiO_2_ 0.8, Horowitz quotient of 75), an acute respiratory distress syndrome (ARDS) was diagnosed, based on the Berlin definition.

During positioning, the patient coughed (TV 139 ml, PIP 37 cm H_2_O, AP mean 16 cm H_2_O) and received 50 mg propofol (i.v.). During cervical lymph node dissection and thymus removal, the care team noted a gurgling and bubbling sound radiating from the thorax. We checked the cuff pressure and tube position using a video laryngoscope, but no abnormalities were found. Three hours into the surgery the thyroid was unroofed and the thyroid ligament was dissected. In addition, all the lymphatic tissue from the carotid and innominate artery towards the midline was cleared out. In addition, the esophagus nerve and the side of the trachea from the thyroid cartilage down to the innominate artery just below the sternum were skeletonized. Again, a cycle of gurgling and bubbling of air into the neck occurred and the patient became difficult to ventilate. The patient slightly desaturated (SpO_2_ 91%) and low tidal volume alarms were noticed. The FiO_2_ was set to 1.0 and the patient was manually ventilated. During manual ventilation decreased resistance was noted and TVs were as low as 42 ml, PIPs were as low as 11 cm H_2_O, and AP mean was as low as 7 cm H_2_O. The ETCO_2_ was 30 mmHg due to increased manual ventilation rates (30/min). To check for an airway leak, the surgical team poured normal saline over the surgical field and observed bubbles in the saline but could not identify any lacerations of the trachea or pleural defects. As ventilation remained difficult with TVs constantly dropping and requiring consecutive recruitment maneuvers over the next 30 minutes, a bronchoscopy was performed. The cuff was deflated and the ET tube was withdrawn until the vocal cords became visible. Ventilation was paused and the bronchoscopy was performed which showed a normal looking tracheal and lobar bronchial mucosa. However, the distal trachea and left mainstem bronchus appeared dynamically collapsed. Subsequent intraoperative chest X-ray revealed a small left-sided pneumothorax and a mild pneumomediastinum (Figure 2). The surgeon placed a chest tube to relieve the pneumothorax. Several hours later, the patient once again became difficult to ventilate and a second chest X-ray was performed to verify the correct position of the chest tube. While the left pneumothorax had decreased in size, a new right pneumothorax had developed (Figure 2) and a second chest tube was placed. After parathyroidectomy, near total thyroidectomy, bilateral central neck dissection, and left lateral neck dissection the surgery was aborted after 11.5 hours secondary to recurrent unstable clinical status. The patient was transferred, intubated, and hemodynamically stable, to the intensive care unit. On postoperative day 1, the patient met extubation criteria. Postextubation chest X-ray showed proper chest tube position, but the pneumothoraces had increased (Figure 3). In the hours following extubation, the patient stated shortness of breath and experienced labored breathing and pressure in the chest. The heart rate [104/min] and the systolic blood pressure became elevated [180 mmHg], and the SpO_2_ dropped to 80% with a respirator rate of 41/min, indicating acute hypoxic respiratory failure and requiring reintubation (Figure 4). An echocardiogram showed normal left ventricular function (estimated ejection fraction 55.9%) and a severely dilated right ventricle with mild right ventricular hypertrophy. With poor oxygenation (PaO_2_ 60 mmHg, FiO_2_ 0.8) in the absence of cardiac failure, the care team diagnosed a severe respiratory distress syndrome (ARDS) based on the Berlin definition (PaO_2_/FiO_2_ < 100 and PEEP 5+, Figure 4) [5]. Following intubation sequential bronchoscopies revealed no evidence for a tracheal injury. As hypercarbic respiratory failure worsened (PaCO_2_ 71 mmHg, pH 7.1, RR 35/min, PEEP 20 cm H_2_O, plateau pressure 32 cm H_2_O, Vt 6.5 ml/kg, I : E ratio 1 : 1, and Adaptive Support Ventilation [Hamilton G5]) with SpO_2_s in the 80s (FiO_2_ 1.0, O_2_ saturation mixed venous 55%), the patient was placed on venovenous extracorporeal membrane oxygenation (vvECMO). Over the following three days the patient's respiratory function improved. VvECMO therapy was discontinued and the patient was subsequently extubated. Follow-up echocardiography found normal heart function with an EF of 73%. We discharged the patient from the hospital on postoperative day 12 in good physical condition. Due to posttraumatic stress disorder, the missing right lateral neck dissection was postponed but successfully performed eight months later.

## 3. Discussion

Pneumothorax is a known complication of thyroid surgery, reported as early as 1947 [6]. Nevertheless, only a few cases have been published suggesting a low incidence rate [7–10]. In strong contrast to this, one study reported pneumothorax as a complication of thyroid surgery in 1.53% of the cases [4]. Thus, the literature might underreport this problem, maybe because it bears the stigma of an iatrogenic complication.

In our case, bubbling and gurgling radiating from the thorax suggested either an airway injury or a leak around the endotracheal tube (ETT). Direct laryngoscopy and auscultation ruled out a partial extubation (e.g., caused by head movements during thyroid preparation). As the ETT cuff maintained the applied pressure, a cuff leak seemed unlikely. In fact, the majority of airway leaks are not associated with cuff defects [11]. Thus, the bubbling and gurgling were most likely a sign of an airway leak.

When air enters the thoracic cavity or the mediastinum, pneumothorax and pneumomediastinum develop. This requires a direct communication either between the alveoli and the pleural cavity or between the atmosphere and the pleura [12]. This can result from tracheal perforation, which has been reported to occur in 0.06% of thyroidectomy cases [13]. During thyroid surgery two possible sites of tracheal injury exist: at the isthmus, when the thyroid is separated from the trachea, and at the area where the recurrent laryngeal nerve enters the thyroid cartilage during lateral and posterior dissection [13]. An air leak usually causes self-resolving subcutaneous emphysema or a pneumomediastinum. When deep cervical lymph node dissection is performed, the air can track through the cervical fascial planes and create a pressure gradient causing a pleural injury. Also, an inadvertent violation of the pleura during deep lymph node dissection would allow air to enter the pleural cavity. However, the absence of obvious subcutaneous emphysema and no visible tracheal perforation during bronchoscopy complicated the clinical scenario in our patient.

In our case, the thyroid gland was described as “rock” hard and after dissecting the thyroid, the thyroid ligaments, and the deep cervical lymph nodes, gurgling and bubbling of air into the neck and difficult ventilation occurred. The size of the excised thyroid was 7.2 cm from superior to inferior, 6.0 cm from right to left, and 2.0 cm from posterior to anterior and weighing 35 grams. In addition, the papillary thyroid carcinoma had spread to the thymus and several lymph node levels (levels III–V), with the largest metastatic focus at a level V lymph node (1.3 cm). The relative large size and the changed tissue structure of the thyroid indicate a difficult dissection which is also reflected by the time (3 hours) it took to unroof the thyroid. Thus, the difficult “unroofing” of the thyroid in conjunction with a deep cervical lymph node dissection makes traumatic pneumothorax likely.

However, in our patient pneumomediastinum and pneumothorax could have also developed unrelated to surgery. During intraoperative bronchoscopy, we found dynamic airway collapse at the distal trachea and the left main stem bronchus. This finding can imply preexisting and undetected airway disease [14], including tracheomalacia or chronic obstructive pulmonary disease. Both go along with thinning of the airway walls; thus, high airway pressure (e.g., when the patient coughed during positioning) could have caused an airway leak with subsequent pneumothorax and pneumomediastinum, before surgery even started. The relative large and “rock” hard thyroid gland and airway collapse during bronchoscopy indicate that tracheomalacia could have contributed to the clinical scenario. Since the bronchoscope was advanced during exhalation without any ventilation, the mucosal leak was probably collapsed, missing the mucosal defect. Furthermore, we cannot rule out the possibility that the use of the neural integrity monitor (NIM) tube and electromyogram monitoring contributed to the increased risk of injury. These tubes are slightly stiffer than regular endotracheal tubes and, more importantly, EMG monitoring precludes the use of neuromuscular blocking agents. Although the tube placement was uneventful and proper positioning was confirmed by videolaryngoscopy, negative pressure spontaneous ventilation, such as from coughing, might have caused a breach of the pleura apex leading to a pneumothorax. Nevertheless, in this case, the increase in size of the bilateral pneumothoraces, with persisting pneumomediastinum after extubation, rather strongly suggests a perforation of the airway or the fascial planes undetectable by bronchoscopy. Reintubation and ECMO treatment must have contributed to the resolution of the injury as the patient did not require any further tracheal surgery or neck exploration. It remains remarkable that the intraoperative saline test to detect a perforation seemed positive while the more invasive bronchoscopy revealed a normal mucosa which lulled us into a false sense of security. However, flooding the surgical field with saline and observing bubbles mean that both the pulmonary and parietal pleura have been breached in the presence of positive pressure ventilation. Moreover, the fact that the pneumothoraces occurred serially rather than together is a strong indicator that this was surgical trauma-related and related to on-going dissection.

Why the patient developed ARDS postoperatively is unclear, since common causes such as pneumonia, sepsis, or severe trauma were absent. However, the literature reports one case of unexpected severe respiratory failure in a surgical patient in the presence of dynamic airway collapse undetected prior to surgery [15]. Thus, a combination of a preexisting subclinical airway disease (implied by the dynamic airway collapse) and a significant positive fluid balance (+4000 ml) after surgery could have been the cause for the ARDS in our patient. However, while the positive fluid balance was significant after surgery, the fluid balance was already negative (−650 ml) at the time of reintubation. As the patient did not require blood transfusions, TRALI as cause of ARDS can be ruled out.

In conclusion, we report the first case of acute respiratory failure with ECMO treatment following thyroid surgery, complicated by pneumomediastinum and bilateral pneumothoraces. It highlights that anesthesiologists need to be aware of the anatomical routes and the pathogenesis of air trapping in patients undergoing thyroid surgery in particular in cancer-related bilateral neck dissection. Even in the absence of an obvious airway perforation, the care team should critically evaluate the airway before early postoperative extubation. As also suggested by others [4], any unexplained episodes of oxygen desaturation during surgery or in the recovery room need follow-up chest radiography.
